# Synthesis of a Novel Gold(I) Complex and Evaluation of Its Anticancer Properties in Breast Cancer Cells

**DOI:** 10.2174/0118715206281182231127113608

**Published:** 2024-03-11

**Authors:** Haseeb Ahmad Khan, Anvarhusein Abdulkadir Isab, Abdullah Saleh Alhomida, Mansour Khalil Gatasheh, Ali Rashid Alhoshani, Bashayr Ahmed Aldhafeeri, N Rajendra Prasad

**Affiliations:** 1 Department of Biochemistry, College of Science, King Saud University, Riyadh, 11451, Saudi Arabia;; 2 Department of Chemistry, College of Science, King Fahd University of Petroleum and Minerals, Dhahran, Saudi Arabia;; 3 Department of Pharmaceutical Chemistry, College of Pharmacy, King Saud University, Riyadh, 11451, Saudi Arabia;; 4 Department of Biochemistry and Biotechnology, Faculty of Life Sciences, Annamalai University, Annamalai Nagar, India

**Keywords:** Gold(I) complex, anticancer, cytotoxicity, mitochondrial membrane potential, apoptosis, reactive oxygen species

## Abstract

**Background:**

Platinum complexes are commonly used for cancer chemotherapy; however, they are not only highly-priced but also have various side effects. It is, therefore, important to design affordable anticancer drugs with minimal side effects.

**Methods:**

We synthesized a new gold(I) complex, PF6{(BDPEA)(TPPMS) digold(I)} (abbreviated as PBTDG) and tested its cytotoxicity in MCF-7 breast cancer cells. We also evaluated the effects of PBTDG on mitochondrial membrane potential, generation of reactive oxygen species (ROS) and apoptosis in breast cancer cells.

**Results:**

The IC_50_ values for PBTDG and sorafenib were found to be 1.48 μM and 4.45 μM, respectively. Exposure to PBTDG caused significant and concentration-dependent depletion of ATP and disruption of mitochondrial membrane potential. PBTDG induced 2.6, 3.6, and 5.7-fold apoptosis for 1 µM, 3 µM, and 10 µM concentrations, respectively. The induction of apoptosis by the same concentrations of sorafenib was 1.2, 1.3, and 1.6-fold, respectively. The low concentration of PBTDG (1 µM) induced the generation of ROS by 99.83%, which was significantly higher than the ROS generation caused by the same concentration of sorafenib (73.76%). The ROS induction caused by higher concentrations (5 µM) of PBTDG and sorafenib were 104.95% and 122.11%, respectively.

**Conclusion:**

The lower concentration of PBTDG produced similar cytotoxicity and apoptotic effects that were caused by a comparatively higher concentration of known anticancer drug (sorafenib). The anticancer effects of PBTDG are attributed to its tendency to disrupt mitochondrial membrane potential, induction of apoptosis and generation of ROS. Further studies are warranted to test the anticancer effects of PBTDG in animal models of cancer.

## 1 INTRODUCTION

Cancer remains a predominant global health concern, representing one of the most frequently diagnosed cancers and the second leading cause of cancer-related mortality worldwide [1, 2]. Statistics from the International Agency for Research on Cancer (IARC) indicate that in 2020 alone, approximately 2.3 million women were diagnosed with breast cancer, and around 685,000 deaths were reported [3]. The pathogenesis of breast cancer is multifactorial, and various factors, such as age, genetics, exposure to estrogen, and lifestyle choices, including diet, exercise, and alcohol consumption, contribute to the development of this disease [4]. Recent advances in screening methods, early detection, and improved treatment modalities have resulted in better survival rates for individuals affected with breast cancer [5].

Chemotherapy drugs are powerful medications used to treat cancers by killing or impairing the growth of cancer cells. There are various types of chemotherapy drugs depending on their mode of action. For instance, doxorubicin, which is commonly used to treat breast, lung, ovarian, and thyroid cancers, works by intercalating DNA and inhibiting topoisomerase II enzyme, leading to cell death [6]. The alkylating agent, cyclophosphamide, which is used to treat lymphomas, leukemias, and solid tumors like breast and ovarian cancers, functions by cross-linking DNA strands to prevent cell proliferation [7]. Paclitaxel (Taxol) stabilizes microtubules and prevents their breakdown during cell division, leading to cell death in various malignancies, such as breast, ovarian, and lung cancers [8]. Fluorouracil (5-FU), which is widely used to treat colorectal, breast, head and neck cancers, works by inhibiting thymidylate synthase, a key enzyme involved in DNA synthesis [9].

Platinum-based drugs, such as cisplatin, carboplatin, and oxaliplatin, are widely used chemotherapeutic agents in the treatment of various types of cancers [10]. The potential anticancer mechanisms of these drugs are based on typical interactions between drugs and cellular components, in particular, DNA and various enzymes involved in the synthesis and repair of DNA. Regarding the mechanism related to DNA binding and intra-strand crosslinking, the platinum drugs preferentially bind to purines (adenine and guanine) in DNA molecules, producing intra-strand crosslinks. Because of these crosslinks, the structure of DNA is distorted resulting in obstruction of DNA replication and transcription process [11]. Inhibition of DNA repair enzymes, including nucleotide excision repair (NER) and mismatch repair (MMR) enzymes, is another important mechanism behind the anticancer effects of platinum-based drugs. By inhibiting these enzymes, platinum drugs potentially increase the cytotoxicity linked with the damage of DNA [12]. Another important mechanism is related to the induction of apoptosis due to the accumulation of damaged (unrepaired) DNA from the processes mentioned above, leading to cell cycle arrest and programmed cell death (apoptosis). This process involves the activation of pro-apoptotic proteins like BAX, p53, and caspases [13]. Notwithstanding the clinical effectiveness of these mechanisms, platinum-based drugs are associated with numerous adverse effects due to their non-specific cytotoxicity, such as nephrotoxicity of cisplatin [14] or ototoxicity, which may lead to irreversible hearing loss through damage to inner ear sensory hair cells [15] or peripheral neuropathy, resulting in motor and sensory deficit [16].

Historically, the medical application of gold is tracked back to 2500 BC, as Chinese people relied on gold as an important therapeutic agent [17]. Later on, the antibacterial and anti-tubercular properties of gold compounds were discovered [18]. Gold therapy was also found to alleviate joint pain, which led to the development of auranofin for treating patients with rheumatoid arthritis [19]. In recent years, gold complexes have gained attention due to their potential application in cancer treatment. Initially, the anticancer activity of the gold (I) complex [Au(dppe)^2^]Cl was evaluated in preclinical trials; however, due to certain toxicological concerns, the study was halted [20]. Later on, new kinds of gold (I) complexes with modified ligands with potential anticancer properties were developed [21, 22]. Various studies have been conducted to understand the mechanisms and therapeutic potential of gold complexes in cancer treatment. For instance, Marzano *et al*. [23] demonstrated that gold (I) phosphine complexes can cause apoptosis in cancer cells. Similarly, Ott *et al*. [24] reported that a gold (I) N-heterocyclic carbene complex exhibited cytotoxic activity against human ovarian cancer cells. In addition to inducing apoptosis in cancer cells, research has also focused on the ability of gold (I) complexes to inhibit angiogenesis, a critical component in tumor growth and metastasis. Bertrand *et al*. [25] found that specific gold (I) complexes could prevent vascular endothelial growth factor (VEGF)-induced angiogenesis. Another significant aspect of the antitumor properties of gold (I) complexes is their ability to modulate protein kinases, which play essential roles in regulating cell proliferation and survival pathways. Navarro-Ranninger *et al*. [26] showed that certain gold (I) phosphine-thiolate complexes can effectively inhibit protein kinases involved in tumor progression. Rubbiani *et al*. [27] reported a series of [Au(NHC)Cl] complexes bearing benzimidazole ligands with thioredoxin reductase (TrxR) inhibition activity being an important mode of action.

An important feature of gold (I) complexes is their ability to target cancerous cells without significantly affecting the normal healthy cells [28]. This selective toxicity of gold(I) complexes has partly been attributed to the production of potentially toxic reactive oxygen species (ROS) in cancer cells [29]. The ROS are highly reactive molecules, and free radicals can damage cellular structures, leading to cell death. The generation of ROS by gold (I) complexes not only results in the inhibition of tumor growth but may also prevent metastasis. Previous studies have shown that gold (I) complexes can induce the generation of potentially noxious ROS in cancer cells. Marzo *et al*. [30] showed that gold (I)-N-heterocyclic carbene complexes increased the levels of intracellular ROS in human ovarian cancer cells at 24 h after the treatment. Kim *et al*. [31] synthesized gold(I) complexes bearing chiral or achiral phosphine ligands and, in addition, mononuclear (C^N)-cyclometalated gold(III) bearing chiral or achiral phosphine ligands, which displayed high cytotoxicity in various cancer cell lines by inducing apoptosis through ROS induction. The specific inhibition of TrxR, an enzyme involved in redox regulation and cellular defense against oxidative stress, is crucial in the inflammatory activity reduction by auranofin as well as gold-based complexes [32].

Given the significant impact of this disease, there is a critical need to develop innovative anticancer drugs that offer enhanced efficacy with minimal adverse effects [33]. Continuous effort in researching novel therapeutic approaches is paramount in addressing this ongoing medical challenge. The development of novel metal-based compounds with pharmacological profiles has emerged as a significant objective in contemporary drug design and medicinal chemistry for cancer treatment [34-36]. By exploring the chemical diversity of metal complexes and elucidating their mechanisms of action, researchers strive to design more potent and selective anticancer agents [37]. Praveen *et al*. [38] reviewed the action of different types of gold catalysts on C−H bond reactions and highlighted the catalytic applications of gold(I) and gold(III) species in creating new complexes with specific ligands. Gold catalytic compounds have also been used to synthesize novel complexes with potential cytotoxic activity against cancer cells [39-41]. In this study, we synthesized a novel gold (I) complex and tested its anticancer activity as well as potential mechanisms of action in breast cancer cells.

## 2 MATERIALS AND METHODS

### 2.1 Synthesis of Gold (I) Complex, PBTDG

The gold(I) complex, PBTDG, was prepared by adding 0.127 g (0.5 mmol) AgPF_6_ dissolved in 5.0 mL of ethanol to chlorido[diphenyl(3-sulfonatophenyl)phosphane)]gold(I), sodium salt hydrate (0.2984 g, 0.5 mmol) in 15.0 mL methanol. The mixture was stirred for 30 minutes at room temperature and then filtered to remove the white precipitate of AgCl. To the filtrate, 0.1164g (0.25 mmol) of the bis(2-dicyclohexylphosphino)ethylamine was added. The contents were stirred for an additional 1 hour and filtered. The clear, colorless solution was kept in an undisturbed area. After 3-5 days, white solid material was obtained, which was washed with dichloromethane and diethyl ether three times (5.0 mL). The complex was then recrystallized from acetonitrile solution. The net product was 0.3174g.

### 2.2 Characterization of Gold Complex

We used a Perkin Elmer Series 11 (CHNS/O) Analyzer 2400 for elemental analysis of the gold complex. NMR spectra were recorded on a JEOL-LA 500 NMR spectrophotometer, operating at a magnetic field of 11.74 T and frequencies of 500 and 125.65 MHz, respectively, for ^1^H and ^13^C NMR. For ^1^H NMR, the spectral conditions were 32 k data points, 3.2 s acquisition time, and 5.75 μs pulse width. The ^13^C NMR spectra were obtained with ^1^H broadband decoupling and the following spectral conditions: 32 k data points, 1.0 s acquisition time, 2.5 s pulse delay, and 5.12 μs pulse width. All spectra were recorded at 297 K in CDCl_3_ relative to tetramethyl silane as an internal standard.

### 2.3 Cell Viability Analysis

The human breast cancer cell line, MCF-7, was obtained from the American Type Culture Collection (Rockville, MD, USA). The experimental protocol was approved by the Institutional Review Board (IRB) of King Saud University, Riyadh, Saudi Arabia (Approval No. KSU-SE-20-73). We used the MTT (3-(4,5-dimethylthiazol-2-yl)-2,5-diphenyltetrazolium bromide) assay for cell viability analysis. MCF-7 breast cancer cells were seeded in a cell culture plate (96-well) at 10^4^ cells per well in 200 μl of Dulbecco's modified Eagle medium (DMEM). The cells were treated with Sorafenib and PBTDG at serial concentrations of 0.3, 1.0, 3.0, 10.0, and 30.0 μM for 24 hours. MTT solution (20 μl of 5 mg/mL) was added to each well of the microplate, which was then incubated in a CO_2_ incubator at 37°C for 3 hours. After incubation, the culture medium was removed, 100 µL of isopropanol was added to each well of the microplate and the absorbance was recorded at 570 nm against reagent blank. The cell viability was calculated using the formula:

Cell viability % = 100*(Absorbance sample) / (Absorbance blank)

### 2.4 Intracellular ATP Analysis

The intracellular ATP levels were determined after the exposure of PBTDG and erlotinib (0-100 μM) to MCF-7 cells for 12 h using a colorimetric ATP assay kit (Sigma-Aldrich, USA) as reported earlier [42]. The unknown concentrations of the test samples were calculated using the linear standard curve generated from the concentration of 0-12 nmole ATP.

### 2.5 Mitochondrial Membrane Potential Analysis

We used the Muse MitoPotential Flow Cytometry Kit (Luminex, IL, USA) for measuring mitochondrial membrane potential (MMP). MCF-7 cells were seeded (15000 cells per well) in a 6-well culture plate. The cells were allowed to culture for 24 hours at 37°C and 5% CO_2_ environment and then exposed to Sorafenib or PBTDG at concentrations of 1 and 5 μM for another 24 hours. A negative control (DMSO) was run in parallel. Finally, the cells were stained with Mito Potential reagents provided in the kit, following the manufacturer’s instructions, and then analyzed by flow cytometry.

### 2.6 Apoptosis Analysis

The apoptotic effects of Sorafenib and PBTDG were evaluated on MCF-7 breast cancer cells, using Muse® Annexin V Live & Dead Cell Kit (Luminex, IL, USA). The cells were seeded in 6-well plates at 15000 cells per well and the plates were incubated for 24 hours at 37°C and 5% CO_2_ environment. Then, the cells were exposed to sorafenib and PBTDG at concentrations of 1.0, 3.0, and 10.0 μM for 24 hours. For negative control, a diluted solution of DMSO was used so that the final DMSO concentration in each well was <0.1%. The cells were then stained with Annexin V-FITC and Dead Cell reagents according to the manufacturer’s instructions. The percentage of apoptotic cells was determined by flow cytometry.

### 2.7 Reactive Oxygen Species Analysis

For the analysis of reactive oxygen species (ROS), MCF-7 cells were seeded (1.5x10^4^ cells/well) in a 6-well plate and allowed to grow for 24 h at 37°C under the environment of 5% CO_2_ and 95% humidity. After incubation, MCF-7 cells were treated with the gold complex PBTDG and sorafenib at final concentrations of 1 and 5 μM for 24 h. DMSO served as a negative control. The final DMSO concentration in each well was less than 0.1%. After harvesting the cells, they were stained with Muse® Oxidative Stress Kit (Luminex, IL, USA) following the manufacturer’s instructions. The percentage of cells with oxidative stress was measured by flow cytometry analysis.

### 2.8 Statistical Analysis

Data were analyzed by one-way ANOVA followed by Dunnett’s multiple comparison test. *P* values <0.05 were considered as statistically significant.

## 3 RESULTS

The structure of the gold(I) complex PBTDG is shown in Fig. (**1**). The ^1^H NMR chemical shifts and ^13^C NMR chemical shifts of the PBTDG complex are given in Tables **1** and **2**, respectively. The P(CH) protons of the cyclohexyl groups were detected near 1.38 ppm. The signal for the N-H proton was found at the most downfield position (3.19 ppm) of the aliphatic region. The remaining protons of the cyclohexyl part resonated between 1.26 – 1.91 ppm. The aromatic protons resonate between 7.56-8.53 ppm (Table **1**). In the ^13^C NMR spectrum of the gold complex PBTDG, downfield shifts were observed in the cyclohexyl group resonances with respect to the free ligand. The C–N (C1) appeared at the most downfield position at (45 ppm). The C–P (C2) resonance shifted upfield (25.7 ppm), indicating the coordination of diphosphanes. The rest of the aromatic carbon’s peaks were in accordance with the structures of the ligand (Table **2**). In the ^31^P NMR spectrum of PBTDG, the resonances for the phosphane ligand were observed significantly downfield (39.9, 49.3 ppm) with respect to the free ligand (-10.40 ppm). The downfield shift was related to the transfer of electron density from phosphorus to gold(I) ion (Table **2**).

The results of cytotoxicity showed a direct relation between cellular death and the concentration of PBTDG and sorafenib, with IC_50_ values of 1.48 μM and 4.45 μM, respectively. At lower concentrations, particularly at 3.0 µM, PBTDG was found to be more toxic than sorafenib for MCF-7 cells, whereas their toxicities were comparable at 30 µM (Fig. **2**).

The intracellular ATP levels were determined in MCF-7 cells following the exposure of different concentrations of PBTDG and positive control, erlotinib (0-100 μM). The control cells (without drug exposure) showed high levels of cellular ATP (Fig. **3**). The concentrations of ATP were depleted after exposure to PBTDG or erlotinib. The drug-induced depletion of ATP was directly proportional to drug concentration (Fig. **3**).

The mitochondrial membrane was significantly depolarized by PBTDG as compared to positive control, sorafenib (Fig. **4**). The depolarization caused by higher concentration (5.0 μM) of PBTDG and sorafenib was found to be 35.4% and 12.0%, respectively. The low concentration (1.0 μM) of PBTDG and sorafenib depolarized the mitochondrial membrane by 14.2% and 2.7%, respectively (Fig. **4**).

The results of apoptosis analysis showed that PBTDG induced 2.6 folds, 3.6 folds, 5.7 folds apoptosis for 1 µM, 3 µM, and 10 µM concentrations, respectively (Fig. **5**). While the induction of apoptosis for sorafenib was found to be 1.2-folds (1 µM), 1.3-folds (3 µM) and 1.6-folds (10 µM). These findings clearly indicate that PBTDG induced significantly higher apoptotic effects as compared to anti-cancer drug, sorafenib (Fig. **6**).

We investigated the effects of PBTDG and sorafenib on the generation of reactive oxygen species (ROS) in MCF-7 cancer cells (Fig. **7**). The low concentration of PBTDG (1 µM) induced the ROS generation by 99.50%, which was significantly higher to the ROS generation caused by 1 µM of sorafenib (73.76%). The ROS induction caused by higher concentrations (5 µM) of PBTDG and sorafenib were 101.48% and 121.78%, respectively (Fig. **8**).

## 4 DISCUSSION

**Fig. (1):**
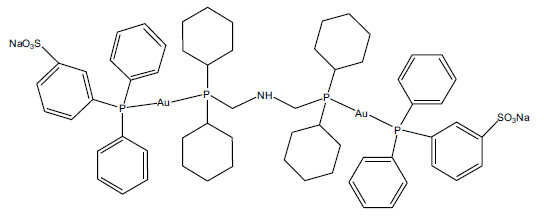
Structure of the gold(I) complex, PF6{(BDPEA)(TPPMS)digold(I)} (abbreviated as PBTDG).

We synthesized a new gold (I) complex, PBTDG (Fig. **1**), which is based on a bimetallic type (digold) linked with two triphenylphosphine (PPh3) ligands. Gold (I) possesses a d^10^ closed-shell kind of configuration and is a soft cation that preferably binds to soft ligands. Owing to this tendency, soft halides, thiolate, and cyanides readily form stable gold complexes, whereas phosphines and other neutral ligands tend to form various kinds of cationic gold complexes [43]. Triphenylphosphine ligands are important phosphorus ligands that have been utilized as gold(I) cation stabilizers. Phosphine ligands not only enhance the solubility but also improve the selectivity and overall performance in catalytic reactions involving gold(I) complexes [44]. Recently, Lu *et al* [45] have reviewed various kinds of gold(I) complexes, classified by their structures, such as NHC–Au(I)–NHC complexes, NHC–Au(I)–phosphine complexes, NHC–Au(I)–thiolate/thiourea/dithiocarbamate complexes, and NHC–Au(I)-halide complexes, where NHC stands for N-heterocyclic carbene.

**Fig. (2):**
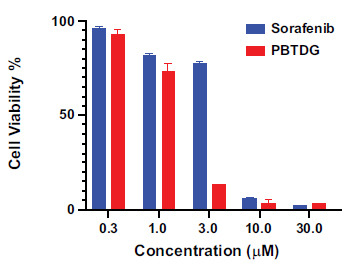
Effects of different concentrations of PBTDG and sorafenib on cytotoxicity in MCF-7 breast cancer cells. MCF-7 cells were seeded in a cell culture plate at 10^4^ cells per well and treated with Sorafenib and PBTDG at serial concentrations of 0.3, 1, 3, 10, and 30 μM for 24 h. MTT solution was added, and the cells were incubated for 3 h. After removing the culture medium, isopropanol was added and the absorbance was recorded at 570 nm for testing the cell viability.

**Fig. (3):**
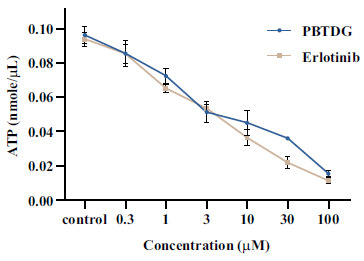
Effects of different concentrations of PBTDG and erlotinib on cellular ATP levels in MCF-7 breast cancer cells. MCF-7 cells were exposed to 0-100 μM concentrations of PBTDG and erlotinib for 12 h, and then the ATP levels were analyzed by using a colorimetric assay kit.

**Fig. (4):**
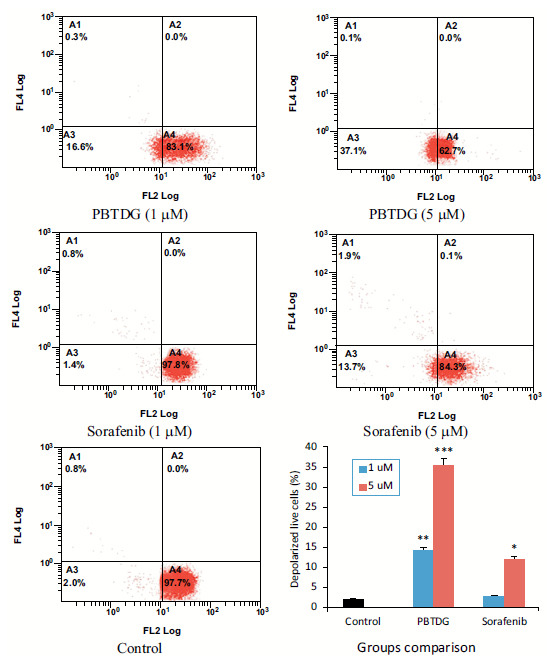
Effects of PBTDG and sorafenib on mitochondrial membrane potential depolarization in cancer cells. MCF-7 cells were seeded (15000 cells per well) in a 6-well culture plate and allowed to grow for 24 h. Then, the cells were exposed to Sorafenib or PBTDG at 1 and 5 μM concentrations for an additional 24 h. The cells were stained and analyzed by flow cytometry. *p < 0.01, **p < 0.001 and ***p < 0.001 *versus* control group.

The gold (I) complex, PBTDG, exhibited anticancer effects in breast cancer cells. The cytotoxic effects of PBTDG were significantly greater than sorafenib, which is a known anticancer drug for solid tumors (Fig. **2**). The IC_50_ value of the gold(I) complex, PBTDG (1.48 μM) in MCF-7 cells, was found to be much lower (more potent) than IC_50_ values of two gold(I) complexes, 4a (7.62 μM) and 4b (4.70 μM), reported earlier by Haque *et al* [46]. The gold (I) complex PBTDG dose-dependently reduced cellular ATP levels (Fig. **3**) and disrupted the mitochondrial membrane potential (Fig. **4**). Gold compounds have been reported to inhibit the proliferation of cancer cells by targeting mitochondrial function. Gold-based compounds, such as auranofin and gold nanoparticles, can disrupt the energy production process within the mitochondria of cancer cells, leading to reduced cell growth and, ultimately, the process of apoptosis (cell death) [47-49]. In addition to auranofin, gold (I) complexes with N-heterocyclic carbene and phosphine ligands have demonstrated promising anticancer activities *in vitro* against various cancer cell lines [50]. These complexes can disrupt mitochondrial function by targeting integral membrane proteins involved in the electron transport chain or inhibiting essential mitochondrial enzymes like topoisomerase II [51]. Zhang *et al*. [52] observed a decrease of the mitochondrial membrane potential (MMP) after 6 h of gold(I) complex treatment, which occurred after the increase of ROS, which was supported by other studies indicating that increased ROS generation can decrease MMP [53].

**Fig. (5):**
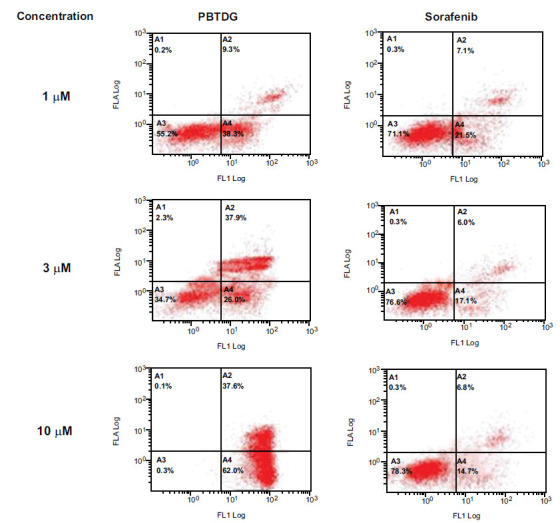
Effects of different concentrations of PBTDG and sorafenib on cellular apoptosis in cancer cells. MCF-7 cells were seeded in 6-well plates (15000 cells per well), and the plates were incubated for 24 h. Then, the cells were exposed to sorafenib and PBTDG at concentrations of 1, 3, and 10 μM for 24 h. The cells were then stained, and the percentage of apoptotic cells was determined by flow cytometry.

**Fig. (6):**
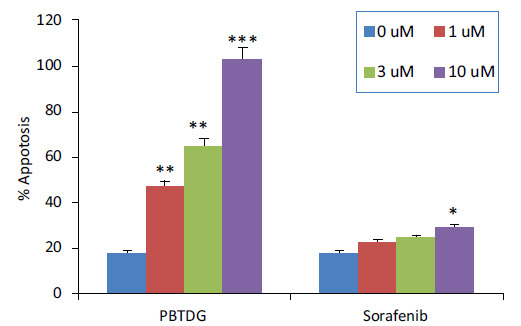
Percent apoptosis (combined early and late apoptosis) induced by different concentrations of PBTDG and sorafenib in cancer cells. *p < 0.05, **p < 0.01 and ***p < 0.001 *versus* the control group (0 μM concentration).

**Fig. (7):**
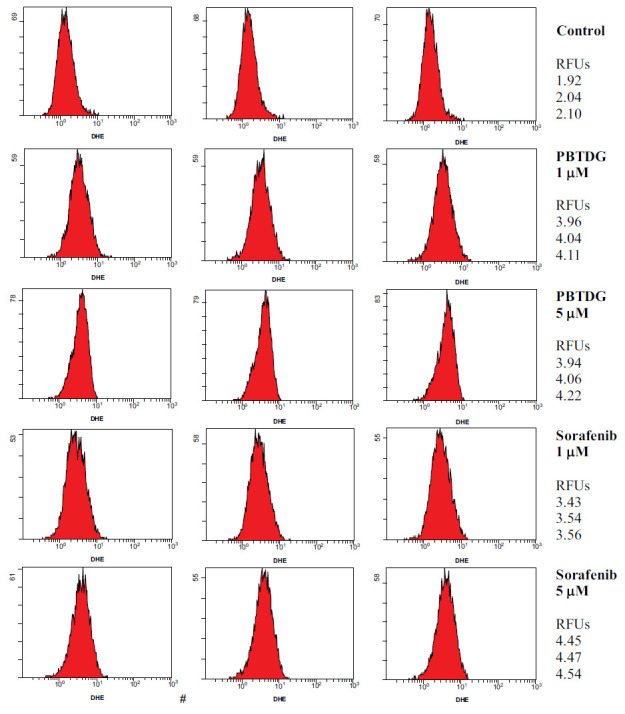
Effects of different concentrations of PBTDG and sorafenib on the generation of reactive oxygen species (ROS) in cancer cells. MCF-7 cells were seeded (1.5x10^4^ cells/well) in a 6-well plate and allowed to grow for 24 h and then treated with PBTDG and sorafenib at final concentrations of 1 and 5 μM for 24 h. The cells were stained, and the percentage of cells with oxidative stress was measured by flow cytometry analysis. The histograms are given for individual tests (N=3 per group). The right column shows the relative fluorescence units (RLUs) for each group.

**Fig. (8):**
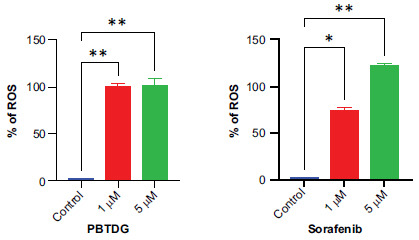
Percent reactive oxygen species (ROS) generation caused by different concentrations of PBTDG and Sorafenib relative to the control group *p < 0.01 and **p < 0.001 *versus* the control group.

PBTDG exerted an anti-proliferative effect by inducing apoptosis in cancer cells (Fig. **5**). We also observed a significant increase in the production of ROS in cancer cells treated with PBTDG (Fig. **8**). Gold (I) complexes are potential anticancer agents due to their unique chemical properties and mechanisms of action. One of the main features of gold (I) complexes is their ability to selectively bind and interact with specific biomolecules. For instance, auranofin, a gold (I)-containing compound, displays anticancer properties by inhibiting the thioredoxin reductase (TrxR) enzyme, essential for maintaining the redox balance in cells. This inhibition leads to oxidative stress, resulting in apoptosis of cancer cells [54]. A series of [Au(PPh3)(alkynyl)] gold complexes reported by Meyer and Coworkers [55] were able to inhibit TrxR and glutathione reductase activities. The cytotoxicity of these gold(I) complexes was significantly reversed when glutathione (GSH) was added at 1 h (before ROS generation) but not after 6 h of incubation (after ROS generation). These alkynyl triphenylphosphine gold (I) complexes have been shown to affect tumor cell metabolism, disrupt mitochondrial respiration and induce antiproliferative effects in tumor cells [55]. Gold (I)-thiourea complex inhibited the TrxR activity *via* gold(I) coordination with the active sites’ cysteine thiol/selenocysteine selenol [56].

It is important to note that cationic gold(I) complexes exhibit comparatively higher cytotoxic activity than the neutral NHCAuX (X = halide) complexes, which can be attributed to the labile Au–X bonds that make these complexes highly sensitive to thiol-containing proteins. Thus, the cationic gold (I) complexes, as reported in this study, are generally more effective than neutral ones [45]. The complex PBTDG is based on phosphine legend. Phosphine has commonly been used in various kinds of gold(I) complexes, such as NHC–Au(I)–phosphine complexes, Alkyne–Au(I)–phosphine complexes, N-Heterocycle-Au(I)–phosphine complexes, phosphine–Au(I)–phosphine complexes, phosphine–Au(I)–thiolate/ thiourea/ dithiocarbamate complexes, and phosphine–Au–halide complexes. Zhang *et al*. [57] synthesized Au(I)–NHC complexes that were able to accumulate in cancer cell lines; of these, complex 39 with triphenylphosphine ligand was found to be highly selective towards cancer cells (MCF-7, A549, HepG2, and CaCo_2_ cell lines) but no obvious suppression of the cell viability of normal 293T cells. Complex 39 was then investigated in MCF-7, A549, HepG2, and CaCo_2_ cell lines and showed similar inhibition. The antitumor effects of complex 39 were attributed to the inhibition of intracellular TrxR activity by this complex [57].

Among the alkyne–Au(I)–phosphine complexes reported by Hikisz and coworkers [58], the highly active complex 74a showed greater antiproliferative activity as compared to auranofin against HepG2, MCF-7 and CCRF-CEM cells with a low IC_50_ value (3.50 µM). In the same study, the highest inhibition of cellular growth was noticed for complexes 74a and 75 in MCF-7 and MDA-MB-231 breast cancer cells, and these complexes caused DNA damage to both solid and leukemic cancer cells [58]. Nisi *et al*. [59] synthesized several alkynyl (triphenylphosphine) gold(I) complexes (numbered as 78–84) carrying differentially substituted propargylic amines. All the complexes showed high toxicities for various cell lines, such as HT-29 (human colorectal adenocarcinoma cell line with epithelial morphology), IGROV1 (human ovarian carcinoma), HL60 (human leukemia), and I407 (normal human epithelial intestinal cell line). Of these, complex 79 was found to be the most active complex in all the tested cell lines, with IC_50_ values ranging between 1.70–7.90 µM [59]. Complexes 83 and 84 were found to be potential inhibitors of TrxR, suggesting that the primary target of binuclear gold complexes is not DNA, but they seem to act by inhibiting TrxR at sub-micromolar concentrations [59]. Marmol *et al*. [60] (reported many alkynyl gold(I)–phosphane complexes (87a–d and 88a–d) that were found to be active against different human cancer cell lines. The complex 88c showed the highest potency with IC_50_ values of 2.33 µM, 7.57 µM and 5.88 µM for CaCo_2_/TC7, MCF-7, and HepG2 cell lines. The coordination of flavones and gold(I) favored the interaction of these complexes with COX-1/2 enzyme as well as the redox enzymes, including TrxR and glutathione reductase, resulting in increased production of ROS and cytotoxicity [60]. Tabrizi and Romanova [61] synthesized a series of gold(I) complexes, of which complex 89 exhibited greater toxicity against MCF-7 (IC_50_ = 1.25 µM), MDA-MB-231 (IC_50_ = 1.97 µM), and HT-29 (IC_50_ = 0.98 µM) cells. This complex was more active than auranofin as it inhibited the activity of TrxR with nanomolar range IC_50_ values and increased the ROS levels to about 5-fold as compared to control [61]. Recently, it has been emphasized for gold compounds to be viable and more effective in cancer treatment, nano-formulation strategies may be essential to improve stability and delivery problems [62].

## CONCLUSION

The anticancer activity of PBTDG at lower concentrations indicates its greater potential for cost-effective cancer chemotherapy. The anti-proliferative capability of PBTDG is associated with the disruption of mitochondrial energy metabolism, generation of ROS, and induction of apoptotic pathways. The selective toxicity of gold complexes against cancer cells, owing to their altered metabolism and higher reliance on mitochondrial energy production compared to healthy cells, has opened promising avenues for novel therapies for cancer. Further studies are warranted to evaluate the therapeutic effectiveness of PBTDG in animal models of cancer.

## Figures and Tables

**Table 1:** ^1^H NMR chemical shifts for gold complex, PBTDG in CDCl_3_.

**C1**	**C2**	**C3**	**C4,4' eq**	**C4,4' ex**	**C5,5' eq**	**C5,5'ex**	**C6,6' eq**	**C6,6' ex**	**Aromatic Hs**	**NH**
3.40	2.51	1.38	2.29	1.22	1.91	1.91	1.74	1.26	7.56, 8.1, 8.53	3.19

**Table 2:** ^13^C and ^31^P NMR chemical shifts for the gold complex, PBTDG, in CDCl_3_.

**C1**	**C2**	**C3**	**C4,**	**C5**	**C6**	**Aromatic Cs**	**^31^P**
45	25.7	34.1	26.5	30.0	29.2	128.5, 129.8, 131.7, 134.1	39.9, 49.3

## Data Availability

The data and supportive information are available within the article.

## LIST OF ABBREVIATIONS

IARC: International Agency for Research on Cancer
MMR: Mismatch Repair
NER: Nucleotide Excision Repair
ROS: Reactive Oxygen Species
